# Association between visceral adiposity index and heart failure: A cross‐sectional study

**DOI:** 10.1002/clc.23976

**Published:** 2023-01-18

**Authors:** Xinyu Zhang, Yijun Sun, Ying Li, Chengwei Wang, Yi Wang, Mei Dong, Jie Xiao, Zongwei Lin, Huixia Lu, Xiaoping Ji

**Affiliations:** ^1^ Key Laboratory of Cardiovascular Remodeling and Function Research, Chinese Ministry of Education, Chinese National Health Commission and Chinese Academy of Medical Sciences, State and Shandong Province Joint Key Laboratory of Translational Cardiovascular Medicine, Department of Cardiology, Qilu Hospital, Cheeloo College of Medicine Shandong University Jinan Shandong China

**Keywords:** heart failure, VAI, visceral adiposity index, visceral fat

## Abstract

**Background:**

Obesity is an important risk factor for heart failure (HF).

**Hypothesis:**

Visceral adiposity index (VAI) is a simple metric for assessing obesity; however, the association between VAI and risk for HF has not been studied.

**Methods:**

A cross‐sectional study involving 28 764 participants ≥18 years of age from the National Health and Nutrition Examination Survey (NHANES), 2009–2018, in the United States was performed. VAI was calculated using body mass index (BMI), waist circumference (WC), triglycerides (TG), and high‐density lipoprotein cholesterol. VAI was analyzed as a continuous and categorical variable to examine its association with HF. Subgroup analysis was also performed.

**Results:**

The highest VAI (fourth quartile [Q4]) was found among males, BMI, systolic and diastolic blood pressure, WC, hypertension, diabetes, liver disease, coronary heart disease, smoking, total cholesterol, and TG. More participants in Q4 took β‐receptor blockers, angiotensin‐converting enzyme inhibitors/angiotensin II receptor blockers/angiotensin receptor‐neprilysin inhibitor, calcium channel blockers, and antidiabetic and antihyperlipidemic medications. Participants with HF exhibited greater VAI. A per‐unit increase in VAI resulted in a 4% increased risk for HF (odds ratio [OR] 1.04 [95% confidence interval (CI) 1.02–1.05]). After multivariable adjustment, compared with the lowest quartile, the OR for Q3 was 1.55 (95% CI 1.24–1.94). Subgroup analysis revealed no significant interactions between VAI and specific subgroups.

**Conclusion:**

VAI was independently associated with the risk for HF. As a noninvasive index of visceral adiposity, VAI could be used for a “one shot” assessment of HF risk and may serve as a novel marker.

## 1 INTRODUCTION

Heart failure (HF) refers to a group of complex clinical syndromes caused by many factors (myocardial dysfunction, valvular diseases, pathological changes in the pericardium and endocardium, and dysfunction of heart rhythm and conduction),^1^ which leads to ventricular systolic or diastolic dysfunction.^2^ In the United States, approximately 6.2 million individuals ≥20 years of age experience HF, with approximately 1 million newly diagnosed cases of HF annually, and the prevalence continues to rise.^3^, ^4^ Data from the European Society of Cardiology show that approximately 1% of patients with HF are <55 years of age and approximately 10% of those with HF are ≥70 years of age.^1^ In developed countries, the incidence rate of HF may decrease after adjusting for age, which may reflect good management of cardiovascular disease (CVD); however, the overall incidence rate of HF is increasing due to aging.^1^ This situation is similar to that observed in developing countries. According to the latest survey results of HF epidemiology released in 2019, the number of chronic HF cases in China is approximately 13.7 million, and its prevalence has increased by 44% in the past 15 years.^5^

Approximately 29%–40% of patients with HF are overweight (body mass index [BMI] 25.0–29.9 kg/m^2^), and 30%–49% are obese (BMI ≥ 30 kg/m^2^). It is noteworthy that obesity is more common in patients with HF with preserved ejection fraction (HFpEF) than in those with HF with reduced ejection fraction (HFrEF), and >80% of HFpEF patients exhibit a BMI in the range of overweight or obesity.^6^, ^7^ However, the relationship between obesity and HF remains controversial. Dunlay et al. demonstrated that obesity, measured according to increased BMI, is a major risk factor for the development of HF.^8^ However, some research appears to highlight the “obesity paradox,”^9^ which means that overweight or grade 1 obesity can also result in a better survival rate for HF. In addition to BMI, visceral adipose tissue (VAT) is an indicator of obesity. Previous studies have shown that patients with HF, especially HFpEF, exhibit higher VAT.^10^, ^11^, ^12^ These studies usually used computed tomography (CT) or magnetic resonance imaging (MRI) to evaluate VAT. The visceral adiposity index (VAI) was calculated using BMI, waist circumference (WC), triglycerides (TG), and high‐density lipoprotein cholesterol (HDL‐c).^13^ Compared to CT or MRI, the calculation of VAI is easier, and more economical and convenient. Previous studies have shown that VAT is associated with diabetes, hyperuricemia, metabolic syndrome, hypertension, atherosclerosis, and vascular calcification.^13^, ^14^, ^15^, ^16^, ^17^, ^18^ However, to our knowledge, the association between VAI and HF has not been studied.

As such, the purpose of this study was to evaluate the association between VAI and HF among middle‐age and elderly participants of the US National Health and Nutrition Examination Survey (NHANES), 2009–2018.

## 2 METHODS

### 2.1 Study population

The NHANES is a nationally representative cross‐sectional study that enrolls participants through a stratified multistage probability and oversampling design that enables weighted analysis that represents the noninstitutionalized, civilian population of the United States (US). Data are released every 2‐year cycle. Each participant represents approximately 50 000 US citizens. All participants provided informed consent before participation, and ethics approval for the study was obtained from the Research Ethics Review Board at the National Centre for Health Statistics, which consisted of a physician, medical and health technicians, and dietary and health interviewers, who conducted surveys through interviews, health measurements, and laboratory tests. An advanced computer system collects and processes all NHANES data. Findings of this survey can be used to determine the prevalence of diseases and their risk factors.

Data for the present study were derived from the 2009–2018 NHANES cycle. In this cohort, 49 693 participants completed the interviews. Participants who were <18 years of age (*n* = 19 341) and those with missing data regarding HF status (*n* = 1588) were excluded. Ultimately, data from 28 764 participants were included in this cross‐sectional study. Participants with HF were defined as those who answered yes to the question, “Has a doctor or other health professional ever told you that you had congestive HF?.” A detailed flow‐diagram illustrating participant selection is presented in Supporting Information: Figure 1. The National Center for Health Statistics Ethics Review Board reviewed and approved the NHANES protocol and all participants provided written informed consent before data collection.

### 2.2 VAI score

VAI score was calculated according to previously reported equations^13^:

For males,

$$\text{VAI}=\text{WC}\left(\text{cm}\right)/\left(39.68+1.88\times \text{BMI}\;\text{kg}/m^{2}\right)\times \left(\text{TG}\left[\text{mmol}/l\right]/1.03\right)\times \left(1.31/\text{HDL}-c\left[\text{mmol}/l\right]\right).$$

For females,

$$\text{VAI}=\text{WC}\left(\text{cm}\right)/(39.58+1.89\times \text{BMI}\;\text{kg}/m^{2})\times (\text{TG}\left[\text{mmol}/l\right]/0.81)\times \left(1.52/\text{HDL}-c\left[\text{mmol}/l\right]\right).$$

NHANES researchers collected anthropometric data (i.e., height, weight, calculated BMI, and WC) and biochemical data (i.e., glycated hemoglobin, direct HDL‐c, and fasting TG) that were used to calculate VAI. A higher VAI score reflected a greater amount of estimated visceral adiposity.

### 2.3 Variables of interest

Potential covariates, including demographics, comorbidities, lifestyle variables, BMI, TG, TC, serum uric acid (UA), estimated glomerular filtration rate (eGFR), and markers of inflammation, were selected based on clinical relevance and statistical significance. The baseline characteristics of the participants, including demographics, comorbidities, and lifestyle information, were obtained using a questionnaire. BMI, WC, and other biochemical parameters were obtained from medical examinations and laboratory assessments performed at the mobile examination center. BMI was calculated as weight (kg) divided by height (m) squared (kg/m^2^). Hypertension was defined as self‐reported physician‐diagnosed hypertension, use of antihypertensive medications, or blood pressure measurement of 140/90 mmHg.^19^ Diabetes mellitus was defined as self‐reported physician‐diagnosed diabetes, taking oral hypoglycemic agents or insulin, fasting glucose level of 126 mg/dl, or plasma glucose level of 200 mg/dl 2 h after an oral glucose tolerance test.^20^ Participants with anemia were defined as those who answered yes to the question, “During the past 3 months, have you been on treatment for anemia?.” Participants with liver disease were defined as those who answered yes to the question, “Has a doctor or other health professional ever told you that you had any kind of liver condition?.” Participants with coronary heart disease were defined as those who answered yes to the question, “Has a doctor or other health professional ever told you that you had coronary heart disease?.” Participants with kidney disease were defined as those who answered yes to the question, “Have you ever been told by a doctor or other health professional that you had weak or failing kidneys? Do not include kidney stones, bladder infections, or incontinence?.” Participants with a history of heart attack were defined as those who answered yes to the question, “Has a doctor or other health professional ever told you that you had a heart attack (also called myocardial infarction)?.” Smokers were defined as those who had smoked at least 100 cigarettes in their lifetimes. eGFR was calculated using the Chronic Kidney Disease Epidemiology Collaboration creatinine equation.^21^ Dietary Inflammatory Index (DII) was calculated based on a 24 h dietary recall interview with each participant.^22^, ^23^ The inflammatory indicator was the neutrophil‐lymphocyte ratio (NLR).^24^, ^25^

### 2.4 Statistical analysis

According to the NHANES analytic guidelines, descriptive results are expressed as weighted mean ± standard error (SE) or median (first quartile, third quartile) (Q2 [Q1, Q3)] for continuous variables and frequency (weighted percentage) for categorical variables. The VAI was analyzed as a continuous and categorical variable (quartiles). Differences in VAI between groups (with and without HF) were tested using the Student's *t*‐test. Differences in characteristics between the groups were tested using the Student's *t*‐test for continuous variables and χ^2^ tests for categorical variables. The odds ratio (OR) and corresponding 95% confidence interval (CI) for HF per unit increase and each quartile, with the lowest quartile as the reference, was estimated using both univariate and multivariate logistic regression models. Tests for linear trends across the VAI categories were conducted using an independent ordinal variable (0, 1, 2, 3) in all models. In addition to the unadjusted model, potential covariates were progressively adjusted in the three models. Model 1 was adjusted for age, sex, and race; model 2 was additionally adjusted for hypertension, diabetes mellitus (DM), smoking, alcohol consumption, coronary heart disease (CHD), kidney disease, and liver disease; and model 3 was further adjusted for eGFR, systolic blood pressure (SBP), diastolic blood pressure (DBP), serum UA, albumin (Alb), hemoglobin (HGB), hematocrit (HCT), and NLR. The restricted cubic spline model was used for dose–response analysis. To explore whether the association between the VAI and HF was modified by sex, age, race, smoking status, and comorbidities, subgroup analyses was performed according to sex (male or female), age group (18–34, 35–54, 55–74 or ≥75 years of age), race, smoking (yes or no), hypertension (yes or no), diabetes (yes or no), CHD (yes or no), liver disease (yes or no), serum UA ( < 350 or ≥350 μmol/L), eGFR (<90 or ≥90 ml/min/1.73 m^2^), and Alb (<40 or ≥40 g/L), and examined the interactions between the stratified variables and VAI using likelihood ratio tests. The “nhanesR” package version 0.9.1.9 was used for data extraction and processing. Free statistics software version 1.4 and the statistical software package R 4.0.1 (R Foundation for Statistical Computing, Vienna, Austria) were used for all analyses. Differences with a two‐tailed *p* < .05 were considered to be statistically significant.

## 3 RESULTS

### 3.1 Characteristics of the study population

Basic characteristics of the study population are summarized in Table 1. In total, 28 764 participants, with a weighted average age of 50 years, were included in this study. According to VAI quartile, participants with the highest VAI (i.e., Q4) had higher values in males, Mexican Americans, other Hispanics, non‐Hispanic whites, BMI, SBP, DBP, WC, hypertension, DM, liver diseases, CHD, smoking, TC, TG, and serum UA. More participants in Q4 took β‐receptor blockers, angiotensin‐converting enzyme inhibitors (ACEIs)/angiotensin II receptor blockers (ARBs)/angiotensin receptor‐neprilysin inhibitor (ARNI), calcium channel blockers (CCBs), antidiabetic medication, and antihyperlipidemic medications. The opposite patterns were observed for non‐Hispanic Blacks, alcohol, and HDL‐c. A comparison of the four groups revealed that sex, race, BMI, SBP, DBP, WC, alcohol, smoking, several diseases (hypertension, DM, HF, liver disease, CHD, heart attack, and kidney disease), relevant test results (TC, HDL‐C, TG, serum UA, eGFR, NLR, DII, lymphocytes (Lym), Alb, HGB, and HCT), and the utilization rate of medications (β‐receptor blocker, ACEI/ARB/ARNI, MRA, CCB, diuretic, antidiabetic, and antihyperlipidemic) were significantly different (*p* < .001).

**Table 1:** Characteristics of the study participants

Variables	All (*n* = 28764)	Q1 (*n* = 7191)	Q2 (*n* = 7191)	Q3 (*n* = 7191)	Q4 (*n* = 7191)	*p*
Age, years	49 (34, 64)	45 (30, 61)	49 (34, 63)	52 (36, 68)	51 (38, 63)	<.001
Gender, no.(%)						<.001
Male	13910 (48.4)	3661 (50.9)	3354 (46.6)	3343 (46.5)	3552 (49.4)	
Female	14854 (51.6)	3530 (49.1)	3837 (53.4)	3848 (53.5)	3639 (50.6)	
Race, no.(%)						<.001
Mexican American	4157 (14.5)	691 (9.6)	1057 (14.7)	1004 (14)	1405 (19.5)	
Other Hispanic	2994 (10.4)	592 (8.2)	762 (10.6)	748 (10.4)	892 (12.4)	
Non‐Hispanic White	11266 (39.2)	2671 (37.1)	2829 (39.3)	2700 (37.5)	3066 (42.6)	
Non‐Hispanic Black	6239 (21.7)	2216 (30.8)	1552 (21.6)	1649 (22.9)	822 (11.4)	
Other Race	4108 (14.3)	1021 (14.2)	991 (13.8)	1090 (15.2)	1006 (14)	
BMI, kg/m^2^	28.5 (24.6, 32.5)	25.1 (22.2, 29.1)	28.0 (24.5, 32.5)	29.3 (26.1, 32.3)	30.4 (27.0, 34.9)	<.001
SBP, mmHg	124.0 (112.0, 132.0)	118.0 (108.0, 130.0)	122.0 (112.0, 134.0)	124.3 (116.0, 132.0)	124.3 (114.0, 134.0)	<.001
DBP, mmHg	70.2 (64.0, 78.0)	70.0 (62.0, 76.0)	70.2 (64.0, 78.0)	70.2 (66.0, 76.0)	72.0 (64.0, 80.0)	<.001
WC, cm	99.5 (89.0, 107.8)	89.0 (80.3, 99.8)	97.7 (88.5, 108.2)	99.5 (97.1, 103.6)	104.7 (95.9, 115.0)	<.001
Hypertension, no.(%)	11989 (41.7)	2318 (32.2)	2842 (39.5)	3243 (45.1)	3586 (49.9)	<.001
Diabetes mellitus, no.(%)	5470 (19.2)	726 (10.2)	1199 (16.9)	1493 (21)	2052 (28.8)	<.001
HF, no.(%)	958 (3.3)	146 (2)	189 (2.6)	340 (4.7)	283 (3.9)	<.001
Anemia, no.(%)	1292 (4.5)	305 (4.2)	304 (4.2)	388 (5.4)	295 (4.1)	<.001
Liver disease, no.(%)	1187 (4.1)	216 (3)	277 (3.9)	295 (4.1)	399 (5.5)	<.001
CHD, no.(%)	1173 (4.1)	196 (2.7)	263 (3.7)	344 (4.8)	370 (5.1)	<.001
Heart attack, no.(%)	1196 (4.2)	202 (2.8)	282 (3.9)	356 (5)	356 (5)	<.001
Kidney disease, no.(%)	997 (3.5)	170 (2.4)	202 (2.8)	350 (4.9)	275 (3.8)	<.001
Alcohol, g/day	0.0 (0.0, 15.4)	0.0 (0.0, 15.4)	0.0 (0.0, 15.4)	15.4 (0.0, 15.4)	0.0 (0.0, 15.4)	<.001
Smoking, no.(%)	12448 (43.3)	2869 (39.9)	3040 (42.3)	3107 (43.2)	3432 (47.7)	<.001
TC, mmol/L	5.0 (4.3, 5.5)	4.6 (4.0, 5.3)	4.8 (4.2, 5.5)	5.0 (4.7, 5.1)	5.2 (4.5, 5.9)	<.001
HDL‐C, mmol/L	1.4 (1.1, 1.6)	1.7 (1.4, 2.0)	1.4 (1.2, 1.6)	1.4 (1.2, 1.4)	1.0 (0.9, 1.2)	<.001
TG, mmol/L	1.5 (0.9, 2.0)	0.7 (0.6, 0.9)	1.2 (1.0, 1.4)	1.7 (1.5, 1.7)	2.7 (2.1, 3.5)	<.001
Serum uric acid, mmol/L	323.8 (267.7, 368.8)	291.5 (243.9, 350.9)	309.3 (261.7, 368.8)	323.8 (309.3, 345.0)	339.0 (279.6, 398.5)	<.001
eGFR, ml/min/1.73 m^2^	94.0 (81.4, 109.4)	100.0 (83.9, 114.8)	96.6 (80.0, 111.7)	94.0 (86.9, 97.6)	94.2 (76.0, 108.2)	<.001
NLR, %	3.3 (2.8, 3.9)	3.2 (2.7, 3.9)	3.3 (2.8, 4.0)	3.3 (3.1, 3.7)	3.3 (2.8, 4.0)	<.001
DII, mg/L	0.9 (−0.1, 2.2)	0.8 (−0.4, 2.1)	1.0 (−0.3, 2.3)	0.9 (0.0, 2.0)	1.1 (−0.2, 2.3)	<.001
Lym,109/L	2.1 (1.7, 2.5)	1.9 (1.5, 2.3)	2.0 (1.6, 2.5)	2.2 (1.9, 2.3)	2.3 (1.8, 2.8)	<.001
Alb, g/L	42.2 (40.0, 44.0)	43.0 (41.0, 45.0)	42.0 (40.0, 45.0)	42.2 (41.0, 43.0)	42.0 (40.0, 44.0)	<.001
HGB, g/L	14.0 (13.1, 14.9)	13.9 (12.9, 14.9)	14.0 (13.0, 15.0)	14.0 (13.4, 14.4)	14.2 (13.2, 15.2)	<.001
HCT, %	41.3 (38.8, 43.9)	41.2 (38.5, 43.9)	41.3 (38.5, 44.2)	41.3 (39.8, 42.5)	41.9 (38.9, 44.7)	<.001
Medications, no.(%)						
β‐receptor blocker	3208 (11.2)	492 (6.8)	697 (9.7)	963 (13.4)	1056 (14.7)	<.001
ACEI/ARB/ARNI	5843 (20.3)	1046 (14.5)	1404 (19.5)	1558 (21.7)	1835 (25.5)	<.001
MRA	235 (0.8)	37 (0.5)	48 (0.7)	83 (1.2)	67 (0.9)	<.001
CCB	2076 (7.2)	404 (5.6)	494 (6.9)	586 (8.1)	592 (8.2)	<.001
Diuretic	3719 (12.9)	628 (8.7)	840 (11.7)	1133 (15.8)	1118 (15.5)	<.001
Antidiabetic	3530 (12.3)	440 (6.1)	757 (10.5)	1013 (14.1)	1320 (18.4)	<.001
Antihyperlipidemic	5957 (20.7)	1069 (14.9)	1417 (19.7)	1648 (22.9)	1823 (25.4)	<.001

Abbreviations: ACEI, angiotensin converting enzyme inhibitor; Alb, albumin; ARB, angiotensin receptor blocker; ARNI, angiotensin receptor‐neprilysin inhibitor; BMI, body mass index; CCB, calcium channel blocker; CHD, coronary heart disease; DBP, diastolic blood pressure; DII, dietary inflammatory index; eGFR, estimated glomerular filtration rate; HCT, hematocrit; HDL‐C, high‐density lipoprotein cholesterol; HF, heart failure; HGB, hemoglobin; Lym, lymphocytes; MRA, mineralocorticoid receptor antagonist; NLR, neutrophil‐lymphocyte ratio; SBP, systolic blood pressure; TC, total cholesterol; TG, triglycerides; WC, waist circumference.

### 3.2 Association between VAI and HF

**Figure 1:**
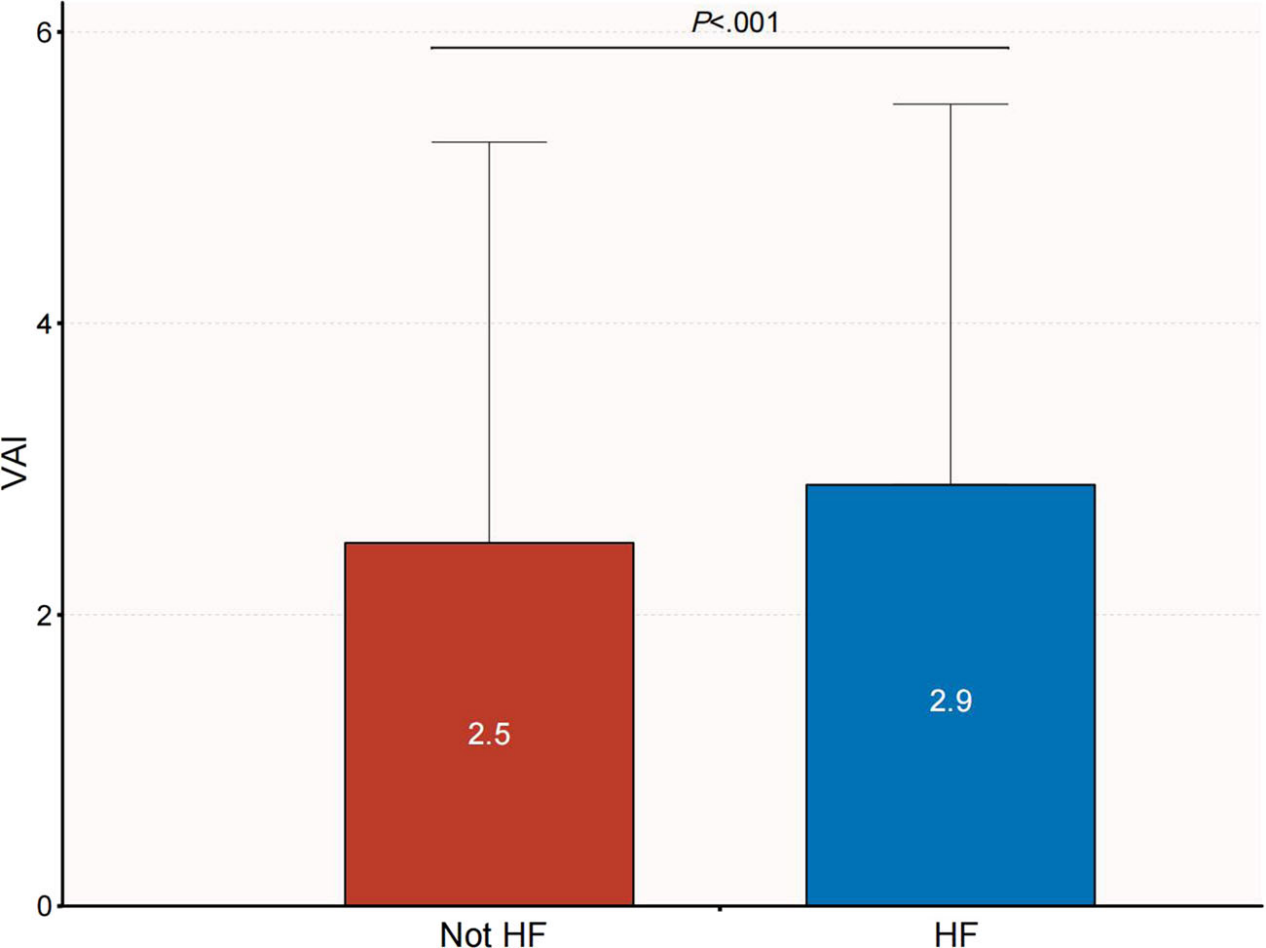
Comparison of VAI between patients with HF and non‐HF. HF, heart failure; VAI, visceral adiposity index.

Differences in VAI between the two groups with and without HF are shown in Figure 1. Results of analysis revealed that participants with HF exhibited a higher VAI than those without HF (*p* < .001).

The relationship between VAI and HF as continuous and categorical variables is shown in Table 2. When VAI was analyzed as a continuous variable, a per unit increase in VAI resulted in a higher risk for HF in the univariate logistic regression model (OR 1.04 [95% CI 1.02–1.05]). The association remained statistically significant in all multivariate logistic regression models after adjusting for several covariates including sex, age, race, hypertension, DM, smoking, alcohol consumption, CHD, kidney disease, liver disease, eGFR, SBP, DBP, UA, Alb, HGB, HCT, and NLR (model 1, OR 1.05 [95% CI 1.03–1.07]; model 2, OR 1.02 [95% CI 1–1.05]; and model 3, OR 1.03 [95% CI 1–1.05]). When VAI was analyzed as a categorical variable, compared with the top VAI quartile, subjects in the third quartile (Q3) had the highest risk for HF (OR 1.55 [95% CI 1.24–1.94]), adjusting for age, sex, race, hypertension, DM, smoking, alcohol, CHD, kidney disease, liver disease, eGFR, SBP, DBP, UA, Alb, HGB, HCT, and NLR.

**Table 2:** Multivariable‐adjusted ORs and 95% confidence intervals of the visceral adiposity index associated with heart failure

	Unadjusted		Model 1^a^		Model 2^b^		Model 3^c^	
	OR (95% CI)	*p*	OR (95% CI)	*p*	OR (95% CI)	*p*	OR (95% CI)	*p*
Continuous per unit increase	1.04 (1.02~1.05)	<.001	1.05 (1.03~1.07)	<.001	1.02 (1~1.05)	.041	1.03 (1~1.05)	.031
Quintiles^d^								
Q 1	1 (Ref)		1 (Ref)		1 (Ref)		1 (Ref)	
Q 2	1.3 (1.05~1.62)	.018	1.26 (1.01~1.58)	.043	1.06 (0.83~1.34)	.641	1.01 (0.79~1.28)	.957
Q 3	2.39 (1.97~2.92)	<.001	1.96 (1.6~2.4)	<.001	1.57 (1.26~1.95)	<.001	1.55 (1.24~1.94)	<.001
Q 4	1.98 (1.61~2.42)	<.001	2.04 (1.65~2.51)	<.001	1.29 (1.02~1.62)	.032	1.19 (0.93~1.51)	.16
*p* for trend	<.001		<.001		.002		.014	

Abbreviations: Alb, albumin; CHD, coronary heart disease; CI, confidence interval; DBP, diastolic blood pressure; DM, diabetes mellitus; eGFR, estimated glomerular filtration rate; HCT, hematocrit; HGB, hemoglobin; NLR, neutrophil‐lymphocyte ratio; SBP, systolic blood pressure; UA, serum uric acid.

^a^ Model 1 adjusted for gender, age and race.

^b^ Model 2 further adjusted for hypertension, DM, smoker, alcohol, CHD, kidney disease and liver disease.

^c^ Model 3 further adjusted for eGFR, SBP, DBP, UA, Alb, HGB, HCT, and NLR.

^d^ The quintile cutoff values of the Visceral Adiposity index are 1.093, 2.041, and 2.676.

**Figure 2:**
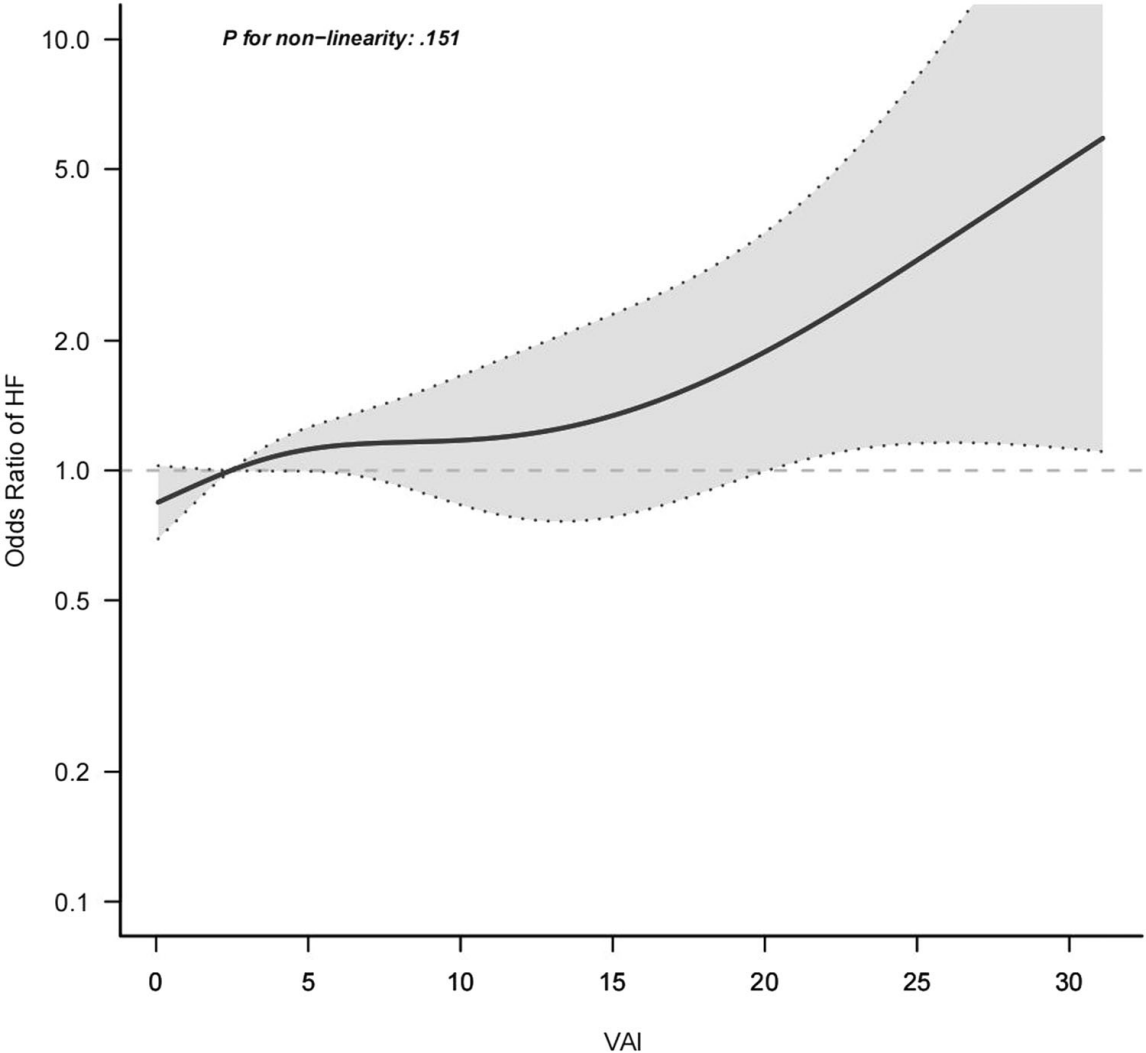
Relationship between VAI and the odds ratio of HF. HF, heart failure; VAI, Visceral adiposity index.

There was a linear relationship between VAI and the OR for HF in Model 3 (*p* for nonlinearity, .151), which used the restricted cubic spline model (Figure 2).

### 3.3 Subgroup analysis

**Figure 3:**
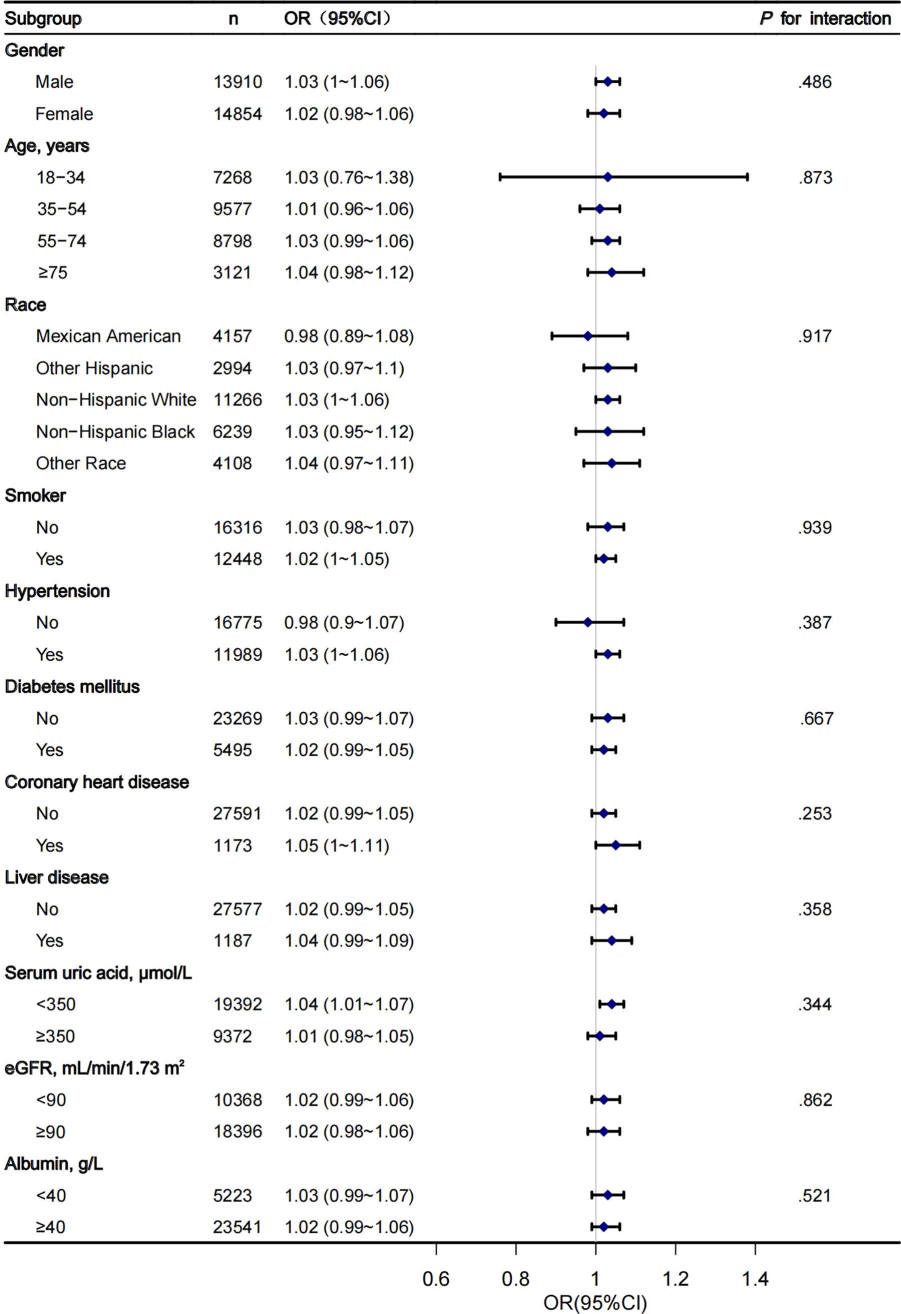
Subgroup analysis of the association between VAI and HF. CI, confidence interval; eGFR, estimated glomerular filtration rate; HF, heart failure; OR, odds ratio; VAI, Visceral adiposity index.

Sex, age, race, smoking status, hypertension, DM, CHD, liver disease, serum UA, and eGFR were used as stratification variables to observe the effect size trend, and a Forrest plot of data was generated (Figure 3). All associations were positive in the different subgroups, except for Mexican Americans and participants without hypertension. There were no significant interactions between VAI and sex, age, race, smoking status, hypertension, DM, CHD, liver disease, serum UA, eGFR, or albumin.

## 4 DISCUSSION

Using data from a representative national sample of middle‐age and elderly populations in the US, we found that VAI was associated with HF after adjustment for other covariates, exhibiting a near linear dose–response relationship. Subgroup analysis makes it possible to better understand VAI and HF in different populations, suggesting that the direction of relationships between the VAI and HF in different subgroups was consistent with that in the total study population.

The overall prevalence of HF in our study was approximately 2.4%, which is consistent with previously published data.^3^, ^4^ Previous studies have reported an association between obesity and HF,^26^, ^27^, ^28^ and determined that obesity is the main risk factor for hypertension, CVD, and left ventricular hypertrophy (LVH), which are strong risk factors for the development of HF.^29^, ^30^ In the Framingham Heart Study, which included 5881 participants, after adjusting for some risk factors, each unit increase in BMI increased the incidence of HF by 5% in males and 7% in females, and the risk for HF increased across the BMI range.^26^ The Physicians’ Health Study, which included 21 094 males (mean age, 53 years) without known CHD at baseline, demonstrated that every 1 kg/m^2^ increase in BMI was associated with an 11% increase in HF risk, and obese participants had a 180% increase in HF risk.^31^ A study involving 59 178 Finnish participants 25–74 years of age who had no HF at baseline reported that the multivariable adjusted risk ratio for HF was highest in the high BMI group (>30 kg/m^2^) among men and women, and abdominal obesity was associated with a greater risk for HF in men and women.^28^

However, obesity includes both overall and abdominal obesity. Different obesity phenotypes may lead to different incidence, mortality, and treatment outcomes of HF.^32^ BMI can be used as an indicator of overall obesity; however, for individuals with simple abdominal obesity, BMI may not be the best indicator, and there is even “normal weight obesity” in the population. Even for normal‐weight individuals, the risk for CVD may be higher among those with high WC. WC, an indicator of abdominal fat, is associated with cardiac metabolic diseases and CVD and can predict mortality.^33^, ^34^ Therefore, other indicators are needed to evaluate abdominal obesity, among which VAT is one. Rao et al. demonstrated that VAT was independently associated with hospitalization for HFpEF in individuals without baseline CVD.^35^ Selvaraj et al. suggested that patients with HFpEF had significantly increased pericardial and subcutaneous fat thicknesses compared to patients without HF.^12^ Sorimachi et al. reported that female HFpEF patients had a higher VAT, and the accumulation of excess VAT played an important role in the pathophysiology of female HFpEF patients.^11^ In these studies, the VAT was measured using abdominal CT or MRI. These methods are accurate but have high cost and low efficiency, and are rarely used in clinics. VAI can be calculated by measuring WC, height, weight, and TG and HDL‐c levels in the blood. The clinical operation is simple and the data are easy to obtain. Previous studies have concluded that VAI is associated with diabetes, hyperuricemia, metabolic syndrome, hypertension, atherosclerosis, and vascular calcification.^13^, ^14^, ^15^, ^16^, ^17^, ^18^ However, to the best of our knowledge, results from previous studies investigating the correlation between VAI and HF are limited. A cohort study of 116 patients 35–80 years of age, who were hospitalized for aggravated HF between 2011 and 2013, demonstrated that VAI may be a good predictor of mortality in patients with ischemic heart failure, and that patients with higher VAI had a better survival prognosis.^36^ However, this study only examined the relationship between VAI and mortality in patients with ischemic heart failure, and did not study the relationship between VAI and the prevalence of HF.

As expected, there was a linear relationship between VAI and the OR for HF. In terms of pathophysiological mechanism, VAT can induce cardiomyocyte hypertrophy, lead to myocardial fibrosis, and activate inflammatory pathways related to macrophage infiltration and cytokine gene expression. Excessive VAT accumulation may lead to higher circulating blood volume and more local and systemic atherogenic inflammatory factors. It may also increase the risk for stroke, increase heart wall pressure and myocardial injury, lead to left ventricular remodeling and, eventually, cause HF.^37^, ^38^, ^39^

To the best of our knowledge, this is the first study to examine the association between VAI and HF in a large and representative national sample of adults in the US. Our study had the advantages of rigorous study protocols and quality controls, a large representative sample, and available data on many vital covariates by integrating the NHANES data. Nevertheless, this study had some limitations. First, the NHANES does not collect echocardiography and N‐terminal pro brain natriuretic peptide (NT‐proBNP) data from participants. Participants with HF were defined as those with self‐reported physician‐diagnosed HF. The same situation also occurs in hypertension, DM, anemia, liver disease, CHD, kidney disease, and a history of heart attack. Second, this was a cross‐sectional study that did not include follow‐up data. The changes in VAI and the risk for HF over time are unclear. Our study design did not permit identification of a causal association between VAI and HF during the study period. Third, it did not distinguish between the types of HF in participants and could not evaluate whether VAI has a different relationship with different types of HF.

## 5 CONCLUSION

Results of the present study revealed that VAI was independently associated with the risk for HF. More simply stated, noninvasive scores of visceral adiposity permitted a simple noninvasive “one shot” assessment of HF risk(s). In view of the increasing prevalence and enormous health burden of HF, individuals with high VAI warrant greater attention to prevent HF. As such, its potential use as a novel marker of HF risk merits further investigation.

## CONFLICT OF INTEREST

The authors declare no conflict of interest.

## Supporting information

Supporting information.

## Data Availability

The data that support the findings of this study are openly available in the National Health and Nutrition Examination Survey (NHANES) at https://www.cdc.gov/nchs/nhanes/index.htm.
