# Supplementary figures and images for: Association between visceral adiposity index and heart failure: A cross‐sectional study
Source: Clin Cardiol. 2023 Jan 18;46(3):310–9. doi: 10.1002/clc.23976 (PMC10018101; doi:10.1002/clc.23976)

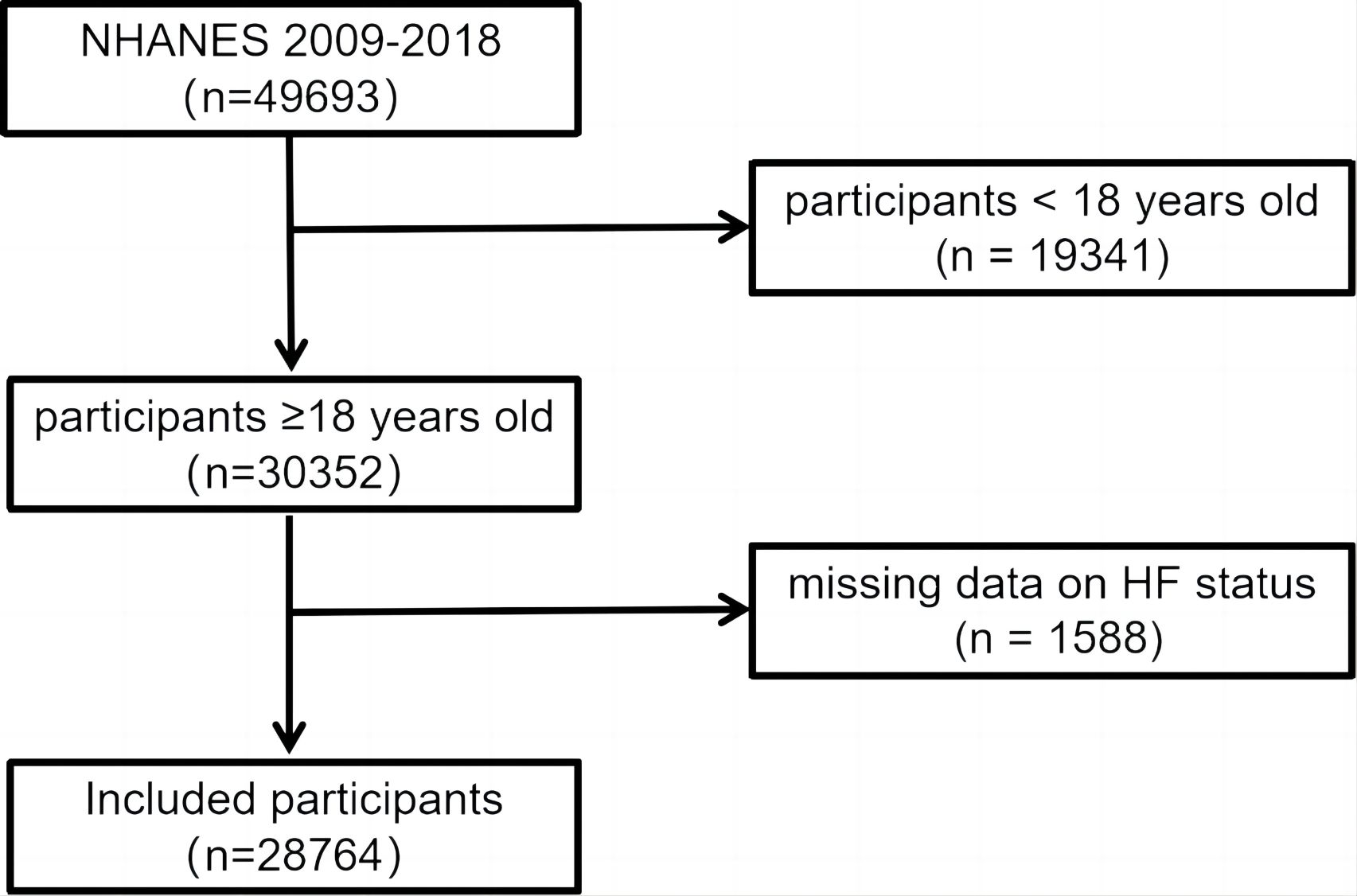

Supplement: Supplementary file 1 — Supporting information. [file CLC-46-310-s001.jpg]
